# Intention and Willingness in Understanding Ritalin Misuse Among Iranian Medical College Students: A Cross-Sectional Study

**DOI:** 10.5539/gjhs.v6n6p43

**Published:** 2014-06-24

**Authors:** Ahmad Ali Eslami, Farzad Jalilian, Mari Ataee, Mehdi Mirzaei Alavijeh, Mohammad Mahboubi, Ali Afsar, Abbas Aghaei

**Affiliations:** 1 Department of Health Education and Promotion, School of Health, Isfahan University of Medical Sciences, Isfahan, Iran; 2 Substance Abuse Prevention Research Center, Kermanshah University of Medical Sciences, Kermanshah, Iran; 3 Social Determinants of Health Research Center, Yasuj University of Medical Sciences, Yasuj, Iran; 4 Health Services Administration, Kermanshah University of Medical Sciences, Kermanshah, Iran; 5 Abadan School of Medical Sciences, Abadan, Iran; 6 Department of Public Health, School of Health, Hamadan University of Medical Sciences, Hamadan, Iran

**Keywords:** Ritalin misuse, college students, behavioral intention

## Abstract

Ritalin misuse can create powerful stimulant effects and serious health risks. The main aim of present study was compared that two cognitive construct (behavioral intention or behavioral willingness) for predicting Ritalin misuse. This cross-sectional study was conducted among 264 Iranian medical college students; participants selected in random sampling, and data were collected by using self-report questionnaire. Data were analyzed by SPSS version 21 at 95% significant level. Our findings showed, the three predictor variables of (1) attitude, (2) subjective norms, and (3) prototype accounted for 29% of the variation in intention and 25% of the variation in willingness to Ritalin misuse. In addition, behavioral intention was a stronger prediction factor compared to willingness for Ritalin misuse, with odds ratio estimate of 1.607 [95% CI: 1.167, 2.213]. There is some support to use the prototype willingness model to design interventions to improve individuals’ beliefs that academic goals are achievable without the misuse of Ritalin.

## 1. Introduction

Methylphenidate is one of the classic amphetamines and stimulates central nervous system (CNS) (Challman & Lipsky, 2000). Due to the rapidly effective quality of these drugs, their behavioral signs and tolerance to them immediately emerge. This issue increases the risk of abuse and dependency in prone cases and because of this; these drugs are categorized among controlled medicine (Sadock et al., 2007). These are used in a wide range to treat attention-deficit hyperactivity disorder (ADHD), attention deficit disorder (ADD) and narcolepsy; because there is no other medicine as effective in treating these disorders (Spencer et al., 2007). Production and prescription of methylphenidate rose significantly in the 1990s, especially in the United States (Biederman et al., 2006). Although Methylphenidate has been used since years ago, and researchers funded people with ADHD do not get addicted to their stimulant medications at treatment dosages (Wilens et al., 2003), bout recently various studies showed its misuse by people for whom it is not a medication (Mannuzza et al., 2008). Ritalin misuse can create powerful stimulant effects and serious health risks (Grabowski et al., 1997). Because the availability is an often an integral part of a drug-abuse potential, and availability of Ritalin is increasing that this situation may lead to dependence (Morton & Stockton, 2000). Numerous studies reporting widespread of misuse of Ritalin among college students has been increasing in recent years (Low & Gendaszek, 2002; Babcock & Byrne, 2000; Teter et al., 2003; Habibzadeh et al., 2010). Student’s use of Ritalin without a prescription often report that they use stimulants for academic reasons (Peterkin et al., 2011). In this regard, the studies showed prevalence of Ritalin misuse among the college students. For example, 34% (GraffLow, 2002), 16% (Babcock, 2000); and additionally, 3% (Teter, 2003) of the students at least used the Ritalin one time in their life (Low & Gendaszek, 2002; Babcock & Byrne, 2000; Teter et al., 2003). Among Iranian college students Habibzadeh (2011) reported that 8.7% of the students in Tabriz University of medical sciences the northeastern of Iran had a history of Ritalin misuse (Habibzadeh et al., 2010).

Although researchers have begun to examine the risk factors and correlates of misuse of Ritalin among college students, bout many questions remain about how reducing and change this behavior or enhancing protective behaviors and it is necessary to understand the factors that cause those (Tonglet et al., 2014). In drug abuse prevention research, it would be useful to know cognitive related factors, such as knowledge and attitudes to predict intention and consequently, behavior (MacKinnon et al., 2001). Theories explain behavior and suggest ways to achieve behavior change, helps describe and identify why a problem exists also predict behaviors under denned conditions and guide the search for modifiable factors like knowledge and attitudes (Glanz et al., 2008). In this regard Prototype/Willingness Model (PWM) which consists of structures, including; attitude, behavioral intention and behavioral willingness that predict the behavior (Gibbons et al., 1998). PWM Innovators claim, there are two pathways to risk distinctions, that it has two important. A reasoned path mediated by (behavioral) intention/expectation and a social reaction path mediated by (behavioral) willingness. A central tenet of the model is a belief that not all health-risk behaviors are intentional, especially among adolescents and young adults (Stock et al., 2013). There is a dichotomy between intentions and willingness in the PWM. According to the model, intentions are plans that have been formulated in order to achieve a particular goal state through certain, instrumental actions. On the other hand, People consider the implications of their actions before they decide to engage or not to engage in a particular behavior. In willingness, does not involve goal states, plans, or instrumental actions. Compared to intentions, BW involves relatively little forethought, which means less consideration of outcomes or consequences. As a result, it also means less acceptance of responsibility for the behavior and its outcomes (Gibbons et al., 1998).

The objective of this study was to determine factors related to Ritalin misuse among college students in Isfahan University of medical sciences, the center of Iran, based on the Prototype/Willingness Model.

## 2. Methods

### Participants and Procedure

This cross-sectional study was conducted on 264 college students in Isfahan University of Medical Sciences, the center of Iran, during 2013. The sample size was calculated at 95% significant level according to the results of a previous study (Habibzadeh et al., 2010), and a sample of 264 was estimated. Of the population of 264, 241 (91.2%) signed the consent form and voluntarily agreed to participate in the study, which has been approved from Isfahan University of Medical Sciences’ institutional review board, and informed consent was obtained from participants.

### Measure

Data collection conducted after receiving approval from the relevant university ethics committee, this project was carried out, and the volunteers were given the self-questionnaire. The variables assessed in this study included three sections. Prior to conducting the main project, a pilot study was carried out. Initially, the relevant questionnaires were administered to 30 students who were similar to study population in order to estimate the duration of the study conduction and to evaluate the reliability of the questionnaire.

**A: Background questions:** Background questions were: age (years), marital status (single or married), live in dormitory (yes or no), alcohol use (yes or no), smoking (yes or no) and drug abuse (yes or no).

**B: Ritalin misuse:** To assess whether or not the student had experimented with Ritalin misuse, we used their responses to three questions, which included 1). “Have you ever used Ritalin?”, 2). “Had you ever Ritalin use at during last year?” and 3). “Had you ever Ritalin use at during last month?” For which the response category was yes or no.

**C: PWM variables:** PWM items were designed based on standard questionnaires applied to substance abuse (Gibbons et al., 1998; Stock et al., 2013; Gerrard et al., 2008; Jalilian et al., 2013; Mirzaei Alavijeh et al., n.d.).

#### Attitudes

The measure of attitude toward Ritalin misuse was responses to eight items, for example “I If Ritalin use, it would help me to better concentrate in studying.” Cronbach’s alpha indicated that the attitude scale possessed good internal reliability (0.83).

#### Subjective Norms

Subjective norm toward Ritalin misuse was responses by using six items, for example: “my friends think Ritalin use for better concentrate in studying is acceptable.” Cronbach’s alpha showed that the scale possessed good internal reliability (0.76).

#### Prototypes

The scale for prototypes of Ritalin abuser contained seven items asking to what extent the presented characteristics (i.e., cool, sociable, half-baked, etc.) would fit the typical peer who Ritalin abuse answers could be given on a five-point scale (1= not at all to 5= very much). Cronbach’s alpha for the prototype scale was (0.75).

#### Behavioral Intention

Behavioral intention was responses to three items, for example: “I intend to Ritalin use in the during exams times”. Cronbach’s alpha for the behavioral intention scale was (0.70).

#### Willingness to Ritalin Misuse

The willingness to Ritalin misuse was assessed by describing a scenario in which participants were asked to imagine themselves at a party with friends and one of those friends would offer them a Ritalin. This was followed by three questions asking the participants how likely it would be that they would (1) accept the Ritalin, (2) say ‘no thanks’ and refuse, and (3) leave the situation Cronbach’s alpha for the behavioral willingness scale was (0.79).

#### Statistical Analysis

Data were analyzed by SPSS version 21 using appropriate statistical tests, including correlation, and linear regression at 95% significant level.

## 3. Result

The mean age of respondents was 23.02 years [95% CI: 22.73, 23.31], ranged from 18 to 29 years. Of the 241 respondents, 6.6% (16/241) were reported had a history of Ritalin misuse in life time. Furthermore, 3.7% (9/241), and 5.8% (14/241) respectively were reported history of Ritalin misuse during at last month and during at last year.

Nearly 21.2% (51/241) had history of cigarette smoking, and 14.9% (36/241) reported drinking alcohol in lifelong as well as 3.7% (9/241) for opium, and 1.7% (4/241) history of methamphetamine use.

11.2% participants (27/241) were married, and 88.8 % (214/241) were single. Most of the students who had a history of Ritalin misuse (13/16 Ritalin abuser) reported to improve academic performance were a main motivation factor for Ritalin misuse.

A series of chi-square tests was performed to assess the relationship between Ritalin misuse and sex, marital status, educational level, living in dormitory, smoking, drug abuse and alcoholic drinking (Table 1). Our findings showed sex, educational level, smoking and alcohol consumption were statistically significant with Ritalin misuse.

**Table 1:** Demographic characteristics of the Ritalin users and Non-users

Variable		Users n (%)	Non Users n (%)	P-value
Sex	Male	13 (12.9%)	88 (87.1%)	0. 001
	Female	3 (2.1%)	137 (97.9%)	

Marital Status	Single	14 (6.5%)	200 (93.5%)	0. 865
	Married	2 (7.4%)	25 (92.6%)	

Educational Level	BSc	3 (2.9%)	101 (97.1%)	0. 041
	MD	13 (9.5%)	124 (90.5%)	

Living in Dormitory	Yes	11 (7.1%)	144 (92.9%)	0. 702
	No	5 (5.8%)	81 (94.2%)	

Smoking	Yes	8 (15.7%)	43 (84.3%)	0. 003
	No	8 (4.2%)	182 (95.8%)	

Drug Abuse	Yes	0 (0%)	9 (100%)	0. 415
	No	16 (6.9%)	216 (93.1%)	

Alcohol Consumption	Yes	8 (22.2%)	28 (77.8%)	0. 001
	No	8 (3.9%)	197 (96.1%)	

Mean (SD) and frequency (number and percent) for response of PWM items shown in Tables 2 and 3. As can be seen in Tables 2 and 3 among the PWM items, believe such as Ritalin use would help me to be more confidence, to be relax, and better concentrate in studying was achieve highest mean among the attitude items. Commonly use of Ritalin among friends and accept friends believe about use of Ritalin was a highest mean among the subjective norms items. Among the prototype items, egocentric was a highest mean. In addition in our study leave the situation was a highest mean among the behavioral willingness items. Furthermore, behavioral intention to Ritalin use during exams times was a highest mean among the behavioral intention items.

**Table 2:** Response of attitude, subjective norms and prototype items among the participants

	Very Little				Very Much	Men (SD)
Attitude						
If I Ritalin use, it help me to feel happy.	91 (37.8 %)	55 (22.8 %)	60 (24.9 %)	27 (11.2 %)	8 (3.3 %)	2.19 (1.15)
If I Ritalin use, it would help me to be relax.	51 (21.2 %)	67 (27.8 %)	68 (28.2 %)	31 (12.9 %)	24 (10 %)	2.62 (1.23)
If I Ritalin use, it would be thinking and my memory to disturbed.	71 (29.5 %)	50 (20.7 %)	71 (29.5 %)	41 (17 %)	8 (3.3 %)	2.43 (1.17)
If I Ritalin use, it would help me to be more time with my friends.	78 (32.4 %)	57 (23.7 %)	73 (30.3 %)	26 (10.8 %)	7 (2.9 %)	2.28 (1.11)
If I Ritalin use, it would help me to better concentrate in studying.	49 (20.3 %)	80 (33.2 %)	62 (25.7 %)	27 (11.2 %)	23 (9.5 %)	2.56 (1.20)
If I Ritalin use, it would be to confusing me.	82 (34 %)	71 (29.5 %)	59 (24.5 %)	20 (8.3 %)	9 (3.7 %)	2.18 (1.10)
If I Ritalin use, it would be addictive me.	85 (35.3 %)	55 (22.8 %)	69 (28.6 %)	28 (11.6 %)	4 (1.7 %)	2.21 (1.10)
If I Ritalin use, it would help me to be More confidence.	46 (19.1 %)	79 (32.8 %)	68 (28.2 %)	24 (10 %)	24 (10 %)	2.58 (1.19)

Subjective norms						
Ritalin use is common among my friends.	49 (2.3 %)	72 (29.9 %)	69 (28.6 %)	34 (14.1 %)	17 (7.1 %)	2.57 (1.16)
Many of students use Ritalin to concentrate better while studying.	66 (27.4 %)	77 (32 %)	72 (29.9 %)	24 (10 %)	2 (0.8 %)	2.24 (0.99)
My friends, think use of Ritalin to concentrate better while studying is appropriate.	100 (41.5%)	43 (17.8 %)	64 (26.6 %)	30 (12.4 %)	4 (1.7 %)	2.14 (1.14)
I accept believe my friends to Ritalin use.	62 (25.7 %)	41 (17 %)	75 (31.1 %)	53 (22 %)	10 (4.1 %)	2.61 (1.20)
If I use Ritalin, my friends will confirm it.	56 (23.2 %)	40 (16.6 %)	76 (31.5%)	62 (25.7 %)	7 (2.9 %)	2.68 (1.17)
I believe It is ethical to use Ritalin without a prescription.	88 (36.6 %)	40 (16.6 %)	63 (26.1 %)	45 (18.7 %)	5 (2.1 %)	2.33 (1.20)

Prototype						
Appealing	107 (44.4%)	55 (22.8 %)	64 (26.6 %)	13 (5.4 %)	2 (0.8 %)	1.95 (1)
Sociable	99 (41.1 %)	64 (26.6 %)	67 (27.8 %)	10 (4.1 %)	1 (0.4 %)	1.96 (0.94)
Half-baked	62 (25.7 %)	59 (24.5 %)	90 (37.3 %)	23 (9.5 %)	7 (2.9 %)	2.39 (1.05)
Confident	71 (29.5 %)	61 (25.3 %)	86 (35.7 %)	20 (8.3 %)	3 (1.2 %)	2.26 (1.01)
Unappealing	51 (21.2 %)	56 (23.2 %)	92 (38.2 %)	29 (12 %)	13 (5.4 %)	2.57 (1.11)
Egocentric	49 (20.3 %)	59 (24.5 %)	94 (39 %)	32 (13.3 %)	7 (2.9 %)	2.53 (1.04)
Cool	69 (28.6 %)	37 (15.4 %)	90 (37.3 %)	35 (14.5 %)	10 (4.1 %)	2.50 (1.16)

**Table 3:** Response of behavioral willingness and behavioral intention items among the participants

	Very Little				Very Much	Men (SD)
Willingness to use Ritalin						
Imagine themselves at a party with friends and one of those friends would offer them a Ritalin. How likely it would be that they would:						
Accept the Ritalin.	121 (50.2%)	33 (13.7 %)	62 (25.7 %)	14 (5.8 %)	11 (4.6 %)	2 (1.18)
Say ‘no thanks’ and refuse.	97 (40.2 %)	61 (25.3 %)	52 (21.6 %)	14 (5.8 %)	17 (7.1 %)	2.14 (1.21)
Leave the situation	88 (36.5 %)	38 (15.8 %)	72 (29.9 %)	19 (7.9 %)	24 (10 %)	2.39 (1.31)

Behavioral Intention to use Ritalin						
I Intend to use Ritalin to concentrate better while studying during semester.	56 (23.2 %)	64 (26.6 %)	63 (26.1 %)	34 (14.1 %)	24 (10 %)	2.61 (1.26)
I intend to Ritalin use during exams times.	60 (24.9 %)	40 (16.6 %)	81 (33.6 %)	42 (17.4 %)	18 (7.5 %)	2.65 (1.22)
I would suggest use of Ritalin to my friends.	64 (26.4 %)	38 (15.8 %)	79 (32.8 %)	43 (17.8 %)	17 (7.1 %)	2.56 (1.24)

Table 4 shows bivariate correlations between the PWM constructs, which were all statistically significant at either .05 or .01 level. The results showed that intention to Ritalin misuse was correlated with the positive attitude towards to Ritalin abuse (r= 0.456), subjective norms (r= 0.285), prototype (r= 0.329), and willingness (r= 0.479).

**Table 4:** Correlation between different components of prototype willingness model

Component	Mean (SD)	X1	X2	X3	X4
X1. Attitude	19.09 (6.31)	1			
X2. Subjective norms	14.60 (4.66)	0.143*	1		
X3. Prototype	16.18 (4.69)	0.270**	0.158*	1	
X4. Willingness	6.53 (3.13)	0.384**	0.287**	0.343**	1
X5. Intention	7.90 (2.60)	0.456**	0.285**	0.329**	0.479**

* p<.05,

** p<.01.

A hierarchical multiple regression analysis was performed to explain the variation in intention and willingness to Ritalin misuse. As can be seen in Table 5, attitude, subjective norms and prototype variables were statistically significant for predicting Ritalin misuse which, they were accounted for 29% of the variation in intention and 25% of the variation in willingness to Ritalin misuse.

**Table 5:** Hierarchical regression analyses predicting willingness and intention to Ritalin abuse by attitude, subjective norms and prototype

Variable	B	SE B	Beta	T	P-value
Willingness					
Attitude	0.145	0.029	0.292	4.974	<0.001
Subjective Norms	0.140	0.038	0.208	3.641	<0.001
Prototype	0.155	0.039	0.232	3.936	<0.001
R2=0.25, F=26.544, p<0.001					

Intention					

Attitude	0.155	0.024	0.375	6.563	<0.001
Subjective Norms	0.112	0.031	0.200	3.594	<0.001
Prototype	0.109	0.032	0.196	3.431	0.001
R2=0.29, F=32.641, p<0.001					

Logistic regression analysis and backward stepwise method was calculated for predictability of PWM variables on Ritalin misuse. As mentioned in statistical analyses, a step-wise model building procedure was conducted and finally on 4rd step the procedure stopped and the best model was selected, among the PWM variables: attitude with odds ratio estimate of 1.213 [95% CI: 1.072, 1.373], and intention with odds ratio estimate of 1.607 [95% CI: 1.167, 2.213], more influential predictor on Ritalin misuse.

**Table 6:** The correlation between different components of prototype willingness model and Ritalin misuse

Variables	Odds Ratio	95 % CI		P value

		Lower	Upper	
Step 1				
Attitude	1.186	1.041	1.352	0.011
Subjective norm	1.009	0.863	1.179	0.911
Prototype	1.104	0.944	1.291	0.214
Willingness	1.121	0.867	1.450	0.382
Intention	1.431	1.011	2.025	0.043
Step 2				
Attitude	1.186	1.041	1.351	0.011
Prototype	1.105	0.945	1.292	0.213
Willingness	1.126	0.877	1.444	0.352
Intention	1.431	1.012	2.025	0.043
Step 3				
Attitude	1.213	1.071	1.373	0.002
Prototype	1.113	0.951	1.304	0.182
Intention	1.531	1.107	2.117	0.010
Step 4				
Attitude	1.213	1.072	1.373	0.002
Intention	1.607	1.167	2.213	0.004

## 4. Discussion

Our study findings suggest that the behavioral intention was a stronger prediction factor compared to the willingness for Ritalin misuse among Iranian medical college students. Furthermore, attitude, subjective norms and prototype variables, were accounted for 29% and 25% of the variation in intention and willingness to Ritalin misuse respectively. It is also important to note that the positive approach our study participants indicated toward their improved academic performance through the misuse of Ritalin without proper attention to the side effects of consumed chemical compounds may put them at greater risk of other seriously harmful drug’s misuse.

The study findings also indicated the prevalence of Ritalin misuse with smoking and alcohol consumption. That is consistent with the results of Stock et al. (2013), and Darredeau et al. (2007) studies.

Another finding of the present study was higher prevalence of Ritalin misuse among male college students. This is in line with the findings of earlier studies investigating the Ritalin misuse among students (Hall et al., 2005; Simoni-Wastila, 2000). In addition, several studies have demonstrated the higher risky behaviors among boys (Costa et al., 2005; Cockerham et al., 2004; Bagheri & Faramarzi, 2012); it appears that because of the least monitoring, higher autonomy and wider social relationships among boys, engaging risky behavior such as drug misuse was higher among them.

As well as, our results indicates was higher prevalence of Ritalin misuse among professional doctorate students compared undergraduate students; this finding could be due to more awareness of Ritalin advantages or more convenient accessibility to medicine among them. However, this has one of the limitations of present study; because of awareness evaluation has not been compared on the field of education in our study.

Our result indicated, Ritalin abuser, have a favorable attitude towards positive outcomes of Ritalin use (e.g., better concentrate in studying); In this regards, Allahverdipour et al. (2009) in their studied showed the relationship between enhancing negative attitudes towards drugs and the reduction of drug abuse. Therefore we recommended designing and implementing training programs to reinforce negative attitudes, and also increasing awareness about side effect of Ritalin misuse among college students. Subjective norms are a person’s perception of other people’s opinion regarding behavioral performance (Ajzen, 1991). In our study commonly use of Ritalin among friends and accept friends believe about use of Ritalin was a highest mean among the subjective norms items. Judson and Langdon (Judson et al., 2009) in their study indicated the relationship between subjective norms and Ritalin misuse. In addition our result showed, egocentric, and leave the situation, respectively was a highest mean among the prototype and willingness items. Though the, leave the situation, is one of method to prevention of drug abuse, but this was better a college students learn the life skills (especially skills against peer pressure and refusal skills). Another findings of present study, was a higher intention to Ritalin use during exams among the participants.

In high-risk behavior research, it would be useful to know how cognitive related factors, are responsible to predict behavior (Allahverdipour et al., 2012). Based on the prototype willingness model (Gerrard et al., 2008) for high-risk behavior predict two hypothesized paths are important, a reasoned path (intention) and a social reaction path (willingness). The main objective of this study was compared behavioral intention and behavioral willingness structure on Ritalin misuse among Iranian medical college students. Our findings showed; behavioral intention was a stronger prediction factor compared to the willingness for Ritalin misuse among college students. In this regards, Litchfield (Litchfield, & White, 2006), stated that willingness further than intention could predict amphetamine. In addition, Hukkelberg (Hukkelberg, & Dykstra, 2009) was reported a similar result for cigarette smoking among adolescents. These results are not consistent with our findings. However, Pomery et al., was reported that up to age 17 or 18, correlations between young people’s willingness and substance abuse are stronger than those between their intentions and use (Pomery et al., 2009). In addition Gerrard et al stated the relation between intention and behavior is relatively low in adolescence, and then it increases with age (Gerrard et al., 2008).

Finally, one of the important reasons to Ritalin misuse among participants of this study was to improve academic performance. This finding is similar to those reported by Teter (Teter et al., 2003) and Habibzadeh (Habibzadeh et al., 2010). As Desantic et al stated (Desantis & Hane, 2010), student’s justifications of this positive reinforcement for the Ritalin misuse. Academic members could have an important role in persuading students to be realistic and to encourage students for engage in appropriate methods study.

## 5. Conclusion

Our findings indicated there is some support to use the prototype willingness model to design interventions to improve individuals’ beliefs that academic goals are achievable without the misuse of Ritalin.

## Limitations

Our study has a few limited, such as: first, data collection was based on self-reporting; this is maybe prone to recall bias; and a second key limitation is the lack of diversity among the participants; we only evaluated the small group of students in medical field’s student in Iran.

## References

[ref1] Ajzen I (1991). The theory of planned behavior. Organizational behavior and human decision processes.

[ref2] Allahverdipour H, Bazargan M, Farhadinasab A, Hidarnia A, Bashirian S (2009). Effectiveness of Skill-Based Substance Abuse Intervention Among Male Adolescents in an Islamic Country: Case of the Islamic Republic of Iran. Journal of drug education.

[ref3] Allahverdipour H, Jalilian F, Shaghaghi A (2012). Vulnerability and the intention to anabolic steroids use among Iranian gym users: An application of the theory of planned behavior. Substance use & misuse.

[ref4] Babcock Q, Byrne T (2000). Student perceptions of methylphenidate abuse at a public liberal arts college. Journal of American College Health.

[ref5] Bagheri P, Faramarzi H (2012). The Dispersal of High Risk Sexual Behaviors in Different Occupations of People Referred to Council Center of Shiraz Medical University. Zahedan Journal of Research in Medical Sciences.

[ref6] Biederman J, Monuteaux M. C, Mick E, Spencer T, Wilens T. E, Silva J. M, Faraone S. V (2006). Young adult outcome of attention deficit hyperactivity disorder: a controlled 10-year follow-up study. Psychological medicine.

[ref7] Challman T. D, Lipsky J. J (2000). Methylphenidate: its pharmacology and uses. In Mayo Clinic Proceedings.

[ref8] Cockerham W. C, Hinote B. P, Abbott P, Haerpfer C (2004). Health lifestyles in central Asia: the case of Kazakhstan and Kyrgyzstan. Social science & medicine.

[ref9] Costa F. M, Jessor R, Turbin M. S, Dong Q, Zhang H, Wang C (2005). The role of social contexts in adolescence: Context protection and context risk in the United States and China. Applied Developmental Science.

[ref10] Darredeau C, Barrett S. P, Jardin B, Pihl R. O (2007). Patterns and predictors of medication compliance, diversion, and misuse in adult prescribed methylphenidate users. Human Psychopharmacology: Clinical and Experimental.

[ref11] Desantis A. D, Hane A. C (2010). Adderall is Definitely Not a Drug: Justifications for the Illegal Use of ADHD Stimulants. Substance Use & Misuse.

[ref12] Gerrard M, Gibbons F. X, Houlihan A. E, Stock M. L, Pomery E. A (2008). A dual-process approach to health risk decision making: The prototype willingness model. Developmental Review.

[ref13] Gibbons F. X, Gerrard M, Ouellette J. A, Burzette R (1998). Cognitive antecedents to adolescent health risk: Discriminating between behavioral intention and behavioral willingness. Psychology and Health.

[ref14] Glanz K, Rimer B. K, Viswanath K (2008). Health behavior and health education: theory, research, and practice.

[ref15] Grabowski J, Roache J. D, Schmitz J. M, Rhoades H, Creson D, Korszun A (1997). Replacement medication for cocaine dependence: methylphenidate. Journal of clinical psychopharmacology.

[ref16] Habibzadeh A, Alizadeh M, Malek A, Maghbooli L, Shoja M. M, Ghabili K (2010). Illicit methylphenidate use among Iranian medical students: prevalence and knowledge. Drug design, development and therapy.

[ref17] Hall K. M, Irwin M. M, Bowman K. A, Frankenberger W, Jewett D. C (2005). Illicit use of prescribed stimulant medication among college students. Journal of American College Health.

[ref18] Hukkelberg S. S, Dykstra J. L (2009). Using the Prototype/Willingness model to predict smoking behaviour among Norwegian adolescents. Addictive behaviors.

[ref19] Jalilian F, Karami-Matin B, Mirzaei Alavijeh M, Ataee M, Mahboubi M, Motlagh F, Agha A (2013). Prevalence and Factor Related to Ritalin Abuse among Iranian Medical College Student: An Application of Theory of Planned Behavior. TERAPEVTICHESKII ARKHIV.

[ref20] Judson R, Langdon S. W (2009). Illicit use of prescription stimulants among college students: prescription status, motives, theory of planned behaviour, knowledge and self-diagnostic tendencies. Psychology Health and Medicine.

[ref21] Litchfield R. A, White K. M (2006). Young adults’ willingness and intentions to use amphetamines: An application of the theory of reasoned action. E-Journal of Applied Psychology.

[ref22] Low K. G, Gendaszek A. E (2002). Illicit use of psychostimulants among college students: a preliminary study. Psychology, Health & Medicine.

[ref23] MacKinnon D. P, Goldberg L, Clarke G. N, Elliot D. L, Cheong J, Lapin A, Krull J. L (2001). Mediating mechanisms in a program to reduce intentions to use anabolic steroids and improve exercise self-efficacy and dietary behavior. Prevention Science.

[ref24] Mannuzza S, Klein R, Truong N, Moulton J, Roizen E, Howell K, Castellanos F (2008). Age of methylphenidate treatment initiation in children with ADHD and later substance abuse: prospective follow-up into adulthood. American Journal of Psychiatry.

[ref25] Mirzaei Alavijeh M, Jalilian F, Movahed E, Mazloomy S, Zinat Motlagh F, Hatamzadeh N (2013). Predictors of Drug Abuse among Students with Application of Prototype/Willingness Model. Journal of Police Medicine.

[ref26] Morton W. A, Stockton G. G (2000). Methylphenidate abuse and psychiatric side effects. Primary care companion to the Journal of clinical psychiatry.

[ref27] Peterkin A. L, Crone C. C, Sheridan M. J, Wise T. N (2011). Cognitive performance enhancement: misuse or self-treatment?. Journal of attention disorders.

[ref28] Pomery E. A, Gibbons F. X, Reis-Bergan M, Gerrard M (2009). From willingness to intention: Experience moderates the shift from reactive to reasoned behavior. Personality and Social Psychology Bulletin.

[ref29] Sadock B. J, Kaplan H. I, Sadock V. A (2007). Kaplan & Sadock’s synopsis of psychiatry: behavioral sciences/clinical psychiatry.

[ref30] Simoni-Wastila L (2000). The use of abusable prescription drugs: the role of gender. Journal of women’s health & gender-based medicine.

[ref31] Spencer T. J, Biederman J, Mick E (2007). Attention-deficit/hyperactivity disorder: diagnosis, lifespan, comorbidities, and neurobiology. Journal of pediatric psychology.

[ref32] Stock M. L, Litt D. M, Arlt V, Peterson L. M, Sommerville J (2013). The Prototype/Willingness model, academic versus health-risk information, and risk cognitions associated with nonmedical prescription stimulant use among college students. British journal of health psychology.

[ref33] Teter C. J, McCabe S. E, Boyd C. J, Guthrie S. K (2003). Illicit methylphenidate use in an undergraduate student sample: prevalence and risk factors. Pharmacotherapy: The Journal of Human Pharmacology and Drug Therapy.

[ref34] Tonglet M, Phillips P. S, Read A. D (2004). Using the Theory of Planned Behaviour to investigate the determinants of recycling behaviour: a case study from Brixworth, UK. Resources, Conservation and Recycling.

[ref35] Wilens T. E, Faraone S. V, Biederman J, Gunawardene S (2003). Does stimulant therapy of attention-deficit/hyperactivity disorder beget later substance abuse? A meta-analytic review of the literature. Pediatrics.

